# Nitrogen and the Baltic Sea: Managing Nitrogen in Relation to Phosphorus

**DOI:** 10.1100/tsw.2001.291

**Published:** 2001-10-26

**Authors:** R. Elmgren, U. Larsson

**Affiliations:** Stockholm University, Department of Systems Ecology, Sweden

**Keywords:** Baltic Sea, nitrogen, phosphorus, eutrophication, nitrogen fixation, management

## Abstract

The Baltic is a large, brackish sea (4 x 105 km2) extending from 54N to ~66N, with a fourfold larger drainage area (population 8 x 107). Surface salinity (2 to 8 PSU) and hence biodiversity is low. In the last century, annual nutrient loads increased to 10 metric tons N and 5 x10 ton P. Eutrophication is evident in the N-limited south, where cyanobacteria fix 2 to 4 x 10 ton N each summer, Secchi depths have been halved, and O_2_-deficient bottom areas have spread. Production remains low in the P-limited north. In nutrient-enriched coastal areas, phytoplankton blooms, toxic at times, and filamentous macroalgae reduce amenity values. Loads need to be reduced of both N, to reduce production, and P, to limit N-fixing cyanobacterial blooms. When large N-load reductions have been achieved locally, algal biomass has declined. So far, P loads have been reduced more than N loads. If this continues, a P-limited Baltic proper may result, very different from previous N-limited conditions. Reaching the management goal of halved anthropogenic N and P loads at minimum cost will require better understanding of biogeochemical nutrient cycles, economic evaluation of proposed measures, and improved stakeholder participation.

## INTRODUCTION

The Baltic Sea, inside the Danish Straits, is the world's second-largest brackish water area, 373,000 km$^2$ (volume 21,000 km$^3$), with a drainage area of $1.6 \times 10^6$ km$^2$ (Fig. 1), and a population of ~$8 \times 10^7$. The Helsinki Convention on the Protection of the Marine Environment of the Baltic Sea Area also covers Kattegat and the Danish Straits. These higher salinity areas with partly different characteristics are excluded here. The Baltic comprises a series of basins, separated by sills, and extends from 54°N to near 66°N, almost to the Arctic Circle. Surface salinity falls successively from 7 to 10 PSU at the Straits to 6 to 8 PSU in the Baltic proper, 5 to 6 PSU in the southern Gulf of Bothnia (Bothnian Sea), and 2 to 4 PSU in the northern Gulf (the Bothnian Bay). Productivity increases with surface salinity. The Baltic proper has a strong halocline at 60- to 80-m depth, below which salinity is 10 to 20 PSU, and a summer thermocline at 15 to 25 m. The deep bottom waters of the Baltic proper are replaced by intermittent inflows of denser water through the Danish Straits. During intervening stagnation periods (normally 2 to 4 years, but up to 16), the bottom waters gradually turn anoxic, while inflows at intermediate depths improve oxygen (O) levels there. Rather few marine and freshwater organisms, and some brackish-water species, live in the low salinity of the Baltic[1].

## BALTIC EUTROPHICATION

Eutrophication has been evident near some large Baltic cities since the early 20$^{th}$ century. The suspicion that anthropogenic eutrophication was also affecting the open Baltic proper arose when Fonselius[2] showed that its $O_2$ deficiency was worse in the 1960s than ever seen before, and its phosphorus (P) concentration had increased. While the short-term $O_2$ dynamics of the Baltic deep basins were clearly driven by the weather, Fonselius[2]

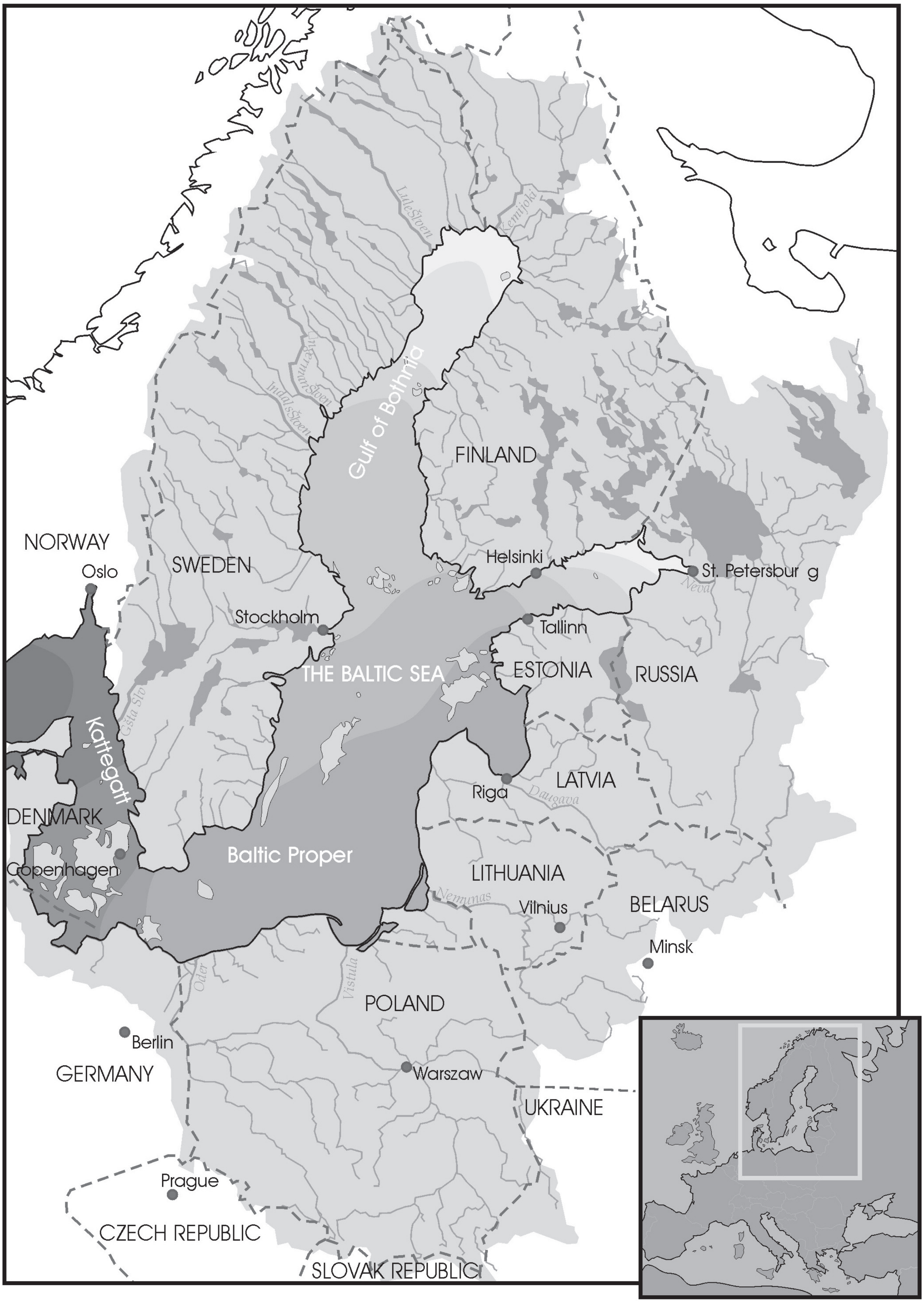

**FIGURE 1.** Map of Baltic Drainage Basin (Source: Stockholm Marine Research Centre). Heavier shading of sea area indicates higher salinity.

thought pollution could be partly responsible for the long-term changes. It was later shown that nitrogen (N) concentrations were also increasing rapidly in most of the Baltic Sea. Winter concentrations of inorganic N and P in surface waters increased rapidly in the Baltic proper from the late 1960s to the early 1980s, peaked in the mid-1980s, and then fluctuated or declined slightly[3]. Concentrations of total P (TP) and total N (TN) in the open Baltic proper surface water likewise increased until the 1980s and have since fluctuated without clear trend[3,4]. Loads were estimated to have increased at least 4X for N, and 8X for P since about 1900[5]. Evidently, the rising nutrient load aggravated the $O_2$ deficiency, by increasing algal production and, hence, sedimentation of organic matter (OM) to the deep water.

## Nature of the Problem

It is now widely agreed that a series of ecological changes in the Baltic proper and the adjacent Gulfs of Finland and Riga have been caused or intensified by rising anthropogenic nutrient inputs after World War II[5,6,7]. Phytoplankton biomass has increased, making the water less transparent, which has reduced submersed vegetation, affected fish foraging, and lowered the value of the coast for tourism, recreation, and nature conservation. N-fixing cyanobacterial blooms, particularly of the toxic *Nodularia spumigena* in the open sea, are a recurring problem. Large bottom areas are covered by $O_2$-deficient waters, which kill fish and invertebrates and change nutrient cycling in ways that worsen eutrophication. Above the $O_2$ deficiency, the biomass of macrobenthos has increased. $O_2$ deficiency has harmed Baltic cod recruitment[8], but positive effects of improved food supply on fish stocks are also found, e.g., for pikeperch in Baltic archipelagos. In the Bothnian Sea, eutrophication[9,10] also affects the large Finnish archipelago, with the rest a transition area to the P-limited, still low-productive, Bothnian Bay in the north.

Limiting nutrients became a practical issue as the need for nutrient removal in coastal sewage treatment plants became clear around 1970, but which nutrient should be removed? In Sweden, P removal had been successful in freshwater and was now applied also to the Baltic, despite indications[11,12,13] that N, rather than P, was limiting phytoplankton in the open Baltic proper. Algal growth potential tests and water chemistry both indicated N as the main limiting nutrient in the open Baltic proper and were later confirmed by a whole-bay experiment[14]. Many similar results eventually led to the requirement in the EU Urban Wastewater Treatment Directive (UWWTD) of N removal in major coastal sewage plants in eutrophication-sensitive coastal areas.

Some still question the limiting role of N in the Baltic proper. Recent demonstrations that both marine and riverine dissolved organic nitrogen (DON) is partly biologically available were taken as proof that phytoplankton can use DON to avoid N limitation after dissolved inorganic nitrogen (DIN) exhaustion by the spring bloom[15]. But while up to half of freshwater-derived DON is biologically available within days in brackish water[16,17], only a few percent of DON present *in situ* in the Bothnian Sea is thus available[18]. It has been calculated that summer DON input by rivers supports at most 5% of summer export production in the Baltic Sea[19]. If, as assumed below, one fifth of atmospheric N input is DON, much of which is bioavailable[20], this could add about 2% more[21]. These calculations, and the regular collapse of the Baltic proper spring phytoplankton bloom once DIN is exhausted (even if phosphate and silicate are still present[14]), make DON use (even if clearly present) unlikely to be a major net N source in the open sea.

Spring N limitation made the regular summer blooms of $N_2$-fixing cyanobacteria in the Baltic proper understandable[22]. Early estimates suggested that they contributed some $13 \times 10^4$ tons of fixed N per year to the Baltic proper, but little to the Gulf of Bothnia[5]. New estimates indicate that cyanobacteria fix 2 to $4 \times 10^5$ tons N per year in the Baltic proper[21,23]. The low phosphate levels common in the Baltic proper during summer blooms of $N_2$-fixing cyanobacteria, as well as phosphate addition experiments, suggest that summer production may sometimes be P-limited, even when the spring bloom is clearly N-limited[14]. Some studies indicate that other phytoplankton remain N-limited even when $N_2$-fixers are P-limited[24,25]. Since filamentous cyanobacteria are rarely used as food, it has been suspected that the N they fix will not stimulate the food chain leading to fish production[26]. It was recently shown, however, that isotopically light N from $N_2$-fixation rapidly permeates the whole Baltic proper food chain, at least up to large zooplankton[27]. This could be due to leakage of fixed N from $N_2$-fixers, which seem to fix more N than needed for their own growth[21]. The presence of small, unicellular N-fixers[23,28] and their use in the food chain, is another possibility. Some recent model results suggest that if cyanobacteria are P-limited, then P may be the ultimate controlling factor for production[30] of offshore phytoplankton, even if phytoplankton are mostly N-limited. Other studies suggest, however, that iron[31] or molybdenum[32,33] may partly limit $N_2$-fixing cyanobacteria in the Baltic proper.

Sediment studies indicate that N-fixing cyanobacteria blooms are almost as old as the present brackish Baltic Sea proper[34], and hence N-limitation and the resulting $N_2$-fixation in summer have been typical features throughout its history. Reducing P inputs only, in the hope of creating a less productive Baltic proper as modeled by Savchuk and Wulff[30], cannot restore this earlier state, but will create a new, P-limited ecosystem if successful. Primary production and sedimentation of $O_2$-consuming OM in a P-limited Baltic would probably be more concentrated to the spring bloom than at present. Since zooplankton largely depends on phytoplankton produced with fixed $N_2$ during its main growth period in the summer[27], this would likely produce less zooplankton and thus fish for the same total primary production.

## Causes of Baltic Eutrophication

Loads of both N and P on the Baltic Sea have undoubtedly increased greatly through human activity[5]. The gradual rise of Baltic nutrient concentrations into the 1980s was generally taken to indicate that loads were still rising. It was therefore a surprise when it was estimated that riverine loads had leveled off by 1970 or soon after and that variability in the riverine loads since 1980 was mainly the result of runoff variations[35]. Estimates of riverine load, coastal point sources, and atmospheric deposition, summing to total external nutrient loads on the Baltic Sea in the 1990s, are given in Table 1. Since published atmospheric deposition estimates for the Baltic Sea include only inorganic N[36], 25% was added in an effort to represent deposition of organic N[37], and 1% of N input was used to estimate atmospheric P input. The molar N:P ratio in the external load is almost 50, well above the Redfield[38] N:P ratio of 16. Even though more of the N load is in organic and relatively refractory form, this massive N excess made the N limitation of phytoplankton in the Baltic hard to explain until huge losses of N through denitrification in the Baltic proper[5,39] were discovered. The factors controlling Baltic denitrification are still insufficiently known (but see Brettar and Rheinheimer[40]).

**TABLE 1**
**Baltic Nutrient Noad (thousand tons/year of N and P)**

| Basin and Area | Riverine Load, Natural + Anthropogenic | Coastal Point Sources | Atmospheric Load, Natural + Anthropogenic | Sum Total | N:P Ratio (molar) |
|---|---|---|---|---|---|
| Gulf of Bothnia* (115,500 km$^2$) | N: 100 P: 5 | N: 10 P: 1 | N: 48 P: <1 | N: 158 P: 6 | 58 |
| Baltic proper (211,100 km$^2$) | N: 363 P: 23 | N: 27 P: 4 | N: 185 P: 2 | N: 575 P: 29 | 44 |
| Gulf of Finland (29,600 km$^2$) | N: 126 P: 6 | N: 31 P: 4 | N: 23 P: <1 | N: 180 P: 10 | 40 |
| Gulf of Riga (16,300 km$^2$) | N: 113 P: 2 | N: 5 P: 1 | N: 11 P: <1 | N: 129 P: 3 | 95 |
| **Baltic Sea total** (373,200 km$^2$) | N: 702 P: 37 | N: 73 P: 9 | N: 267 P: 3 | N: 1042 P: 48 | 48 |

* Gulf of Bothnia = Bothnian Bay + Bothnian Sea. Modified after Elmgren and Larsson[49].

Sources: Riverine load[35], coastal point sources[45], atmospheric N load[53] multiplied by 1.25 to include organic N. Atmospheric P load calculated as 1% of N load.

It has recently been calculated that root-zone leakage of N from Swedish farms may have been as large in the late 19th century as today[41]. The undoubted increase of Swedish riverine loads to the sea may therefore be due mostly to lower losses to denitrification en route from field to sea, due to widespread lowering of lakes, draining of wetlands, and straightening of watercourses[41]. Agriculture fertilizer use in the former communist countries in the Baltic drainage basin[42] fell precipitously after 1989, but with little effect so far on river nutrient loads[43]. A coinciding drop in fertilizer use around the Danube has already led to lower riverine nutrient discharges to the Black Sea[44], so with time an effect is also likely for the Baltic.

Municipal sewage (including individual households) released either directly at the coast or to inland waters is the largest P source to the Baltic. Agriculture is second in importance and industry (mainly the fertilizer and paper industries) is a distant third. Aquaculture is only locally important. Long-range atmospheric transport is far more important for N than for P. Agriculture is clearly still the largest N source via runoff and drainage from fields and through emissions to the air from fertilizers, manure, animals, and farm machinery. Industry and land and sea transport is the second-largest source, through large $NO_x$ air emissions. Municipal sewage is a considerable N source for the Baltic as a whole, particularly near major cities. Industry emissions to water can be locally important, as can aquaculture[7,45,47].

## Current Efforts to Deal with the Problem

Gross pollution near major cities on the Baltic coast was often eliminated in the early 20th century, but nutrient removal only started in the 1970s. Rising environmental awareness around the Baltic Sea[48] led to the 1974 signing of the Helsinki Convention for the Protection of the Marine Environment of the Baltic Sea Area (ratified 1980, updated 1992). In 1988, the Baltic coastal states pledged themselves to halve their 1987 anthropogenic discharges of N and P by 1995. At the 1990 Ronneby Conference, all Baltic coastal states agreed on “Restoring the ecological balance of the Baltic Sea,” with 50% nutrient reduction as a first step.

Since all Baltic coastal states, except Russia, are either EU members or membership candidates, most of the coastal area covered by the Helsinki convention is already subject to EU directives. These include the UWWTD; directives on the quality of waters for bathing, fishery, and mussel culture; the Nitrates Directive; and the recent Water Framework Directive (WFD). In areas designated as sensitive to eutrophication, the UWWTD sets emission standards for all sewage treatment plants larger than 10,000 person-equivalents, even where a major river nearby dwarfs sewage treatment plant emissions. The UWWTD sets no limit on the total quantities discharged and gives no guidance on how to handle nonpoint nutrient sources, which nowadays increasingly dominate the local loads of both N and P to the Baltic coastal zone.

Local regulations can require lower nutrient emissions than the UWWTD and set limits in terms of total element mass emitted. In Sweden, the Himmerfjärden sewage treatment plant (STP) is unique in having emission requirements with latitude to let the plant operate in an adaptive management mode, with the aim of optimizing environmental conditions in receiving waters[49]. Advanced N reduction in the Himmerfjärden STP has recently halved the N load on the receiving coastal bay, where algal biomass, e.g., chlorophyll, and water transparency, e.g., Secchi depth, are known to correlate well with TN concentration[49]. Predictions from such empirical relationships were compared with the measured results of advanced N reduction[49]. During the first two full years, inorganic and TN concentrations declined approximately as predicted, as did mean annual algal biomass, whether estimated as chlorophyll *a* or as carbon (C) (Fig. 2). These results demonstrate that lowering the N load on N-limited coastal areas in the Baltic decreases phytoplankton biomass. As also predicted, the biomass of $N_2$-fixing cyanobacteria in summer increased as inorganic N:P ratios fell, and this change in phytoplankton composition may have been responsible for the absence of a predicted water transparency change in summer[49].

At present, the STP normally removes 80 to 90% of N and about 95% of P, but when monitoring receiving waters indicates the risk of a bloom of $N_2$-fixing cyanobacteria, more N can be released to prevent or reduce the bloom. This was first tried in May to July 2001, and summer biomass of filamentous, $N_2$-fixing cyanobacteria was lower in 2001 than in 2000 and similar to

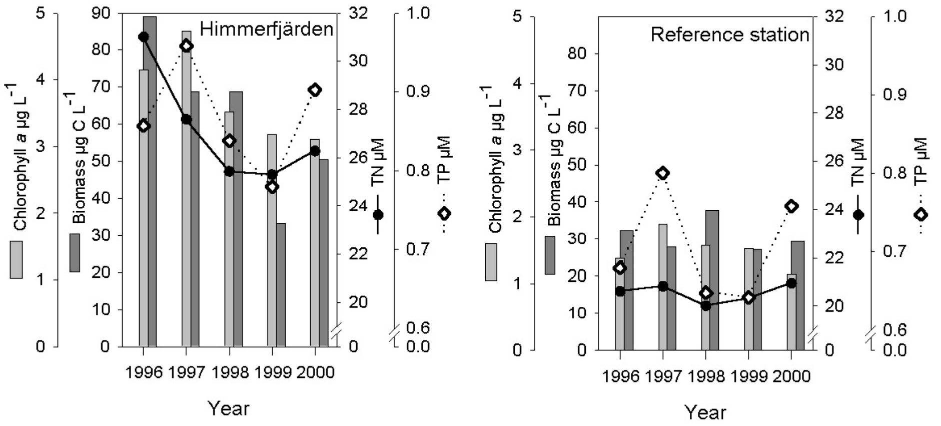

**FIGURE 2.** Left panel: the annual mean phytoplankton biomass (as chlorophyll *a* and C) in the upper mixed layer of Himmerfjärden (Station H4) fell after mid-1997, as N removal in the sewage treatment plant led to lower concentrations of TN and DIN (annual mean about halved, not shown), but not of TP nor of phosphate. Right panel: phytoplankton biomass did not decrease at a nearby reference station in the open coastal zone (Station B1), where neither TN nor TP as annual means changed consistently over time. For location of stations see Elmgren and Larsson[49].

1999. The ruling allowing this could only be obtained after a lengthy political and legal process, also involving local nongovernmental environmental organizations. The Himmerfjärden case is thus of interest both from an ecological and a management point of view.

## Which Are Our Goals?

It is clear that the Water Framework Directive (WFD), based on a watershed approach but also encompassing inner coastal waters, will be very important for the future handling of eutrophication problems in the Baltic coastal zone, and hence, indirectly, also for the open Baltic Sea. Earlier EU directives left little room for meaningful involvement of local stakeholders in managing coastal nutrient discharges. The WFD stresses stakeholder involvement in decision making, rendering it important to describe the management process goals in a way that is understandable to nonspecialists. While there is international agreement to restore the Baltic Sea to an earlier, less polluted state, no clear formulation of the end goal exists. It will thus be impossible to decide if the goal has been attained, or to say which subgoal should have priority, if a conflict arises. When P-load was reduced and water transparency improved in Lake Erie, fishermen protested because the walleye population dropped by over half[50]. Fish stocks are also likely to decrease in the Baltic as nutrient levels fall. The preferred plan of action is thus likely to depend critically on what goals we want to reach—a discussion that has hardly started yet internationally. Sweden has adopted a set of national environmental goals[49], which may act as a starting point for discussions.

## Both N and P, Not Either/Or!

The strategy of the Helsinki Committee (HELCOM) is to reduce both N and P loads, since both concentrations have clearly increased in most of the Baltic Sea. If this diverted funds and efforts from more effective countermeasures, it could indirectly harm the environment. When N concentrations rose in the Bothnian Bay, no eutrophication resulted, since phytoplankton there is P-limited[14]. Removing the locally limiting nutrient will increase the surplus of locally nonlimiting nutrients, which can be exported to areas where they are limiting and will increase production. If these are far away, losses on the way may, however, reduce the problem. It is thus clear that both local and regional effects must be taken into account when managing nutrient discharges to the Baltic coastal zone.

$N_2$-fixing cyanobacteria blooms occur each summer in the Baltic proper, sometimes extending into autumn. They have their own inherent N source and often show indications of P-limitation at their peak[21]. Adding P thus seems likely to enhance such blooms, which can be toxic. Hence the recommendation to also reduce the P load to the Baltic Sea proper and not only that of N, the main limiting nutrient[14]. The higher salinity in the Kattegat seems to limit cyanobacterial blooms[31]. Some assume that any change in P load to the Baltic proper will give a proportional change in the biomass of $N_2$-fixers, and that any N load reduction will be compensated by increased $N_2$-fixation, making N reduction ineffective, e.g., Hellström[29]. A biogeochemical model incorporating similar assumptions predicts that N removal will still reduce algal biomass on a local scale, in the coastal zone, but not in the open Baltic Sea proper, where $N_2$-fixation eventually replaces the removed N[30]. If these assumptions are true, it is, however, difficult to see how the Baltic proper can remain severely N-limited after 7000 years of cyanobacterial summer blooms[34]. It seems that basic chemical differences between fresh and marine waters help limit the development of blooms of $N_2$-fixing cyanobacteria in brackish waters[31,33].

The current management goal of returning the Baltic to a more pristine state requires maintaining the basic N-limitation that causes $N_2$-fixing cyanobacterial blooms. The more strongly N-limited the spring phytoplankton bloom in the Baltic proper, the smaller the export of $O_2$-consuming OM to the bottom water. This should reduce deep-water $O_2$ deficiency, even if total annual net production is maintained by a compensating increase in summer, since less of the summer production sinks out[51]. There are thus good reasons to continue trying to reduce the loads of both N and P on the Baltic Sea.

## Research Needs

More knowledge is needed on how ecological effects in the coastal zone depend on root causes in the watershed, on how best to include stakeholders in nutrient management, and on how to reduce nutrient loads at the lowest possible cost. But even more basic is the need to better understand Baltic nutrient cycling, e.g., how much of the nutrient input to coastal bays and archipelagos actually reaches the open Baltic, and how much is lost en route, through denitrification and deposition in sediments? The Baltic would be awash with nitrates without denitrification, and if it intensified by just 10 to 20%, Baltic eutrophication would be greatly diminished. A central research question is how denitrification will respond to a reduced external load—will it decrease, or will benthic denitrification increase as macrobenthos reinvade formerly anoxic bottom areas? A better understanding of the factors limiting Baltic $N_2$-fixation is needed for management. The effects of N inputs depend on the availability of P, silicate, and iron, and sediment processes are critical in their cycling. Together, these biogeochemical cycles create the unusual Baltic proper ecosystem—brackish and N-limited—yet with large blooms of $N_2$-fixing cyanobacteria. Understanding them is crucial if we are to be able to predict with confidence the long-term effects and cost-effectiveness of different management options for reducing Baltic Sea eutrophication[52].

## CONCLUSIONS

Excess N and P loads from agriculture, sewage, and industry cause eutrophication of the Baltic Sea. N reaches the sea by way of rivers, atmospheric deposition, direct point discharges, and internal $N_2$-fixation, which is stimulated by increased P availability. Efforts to reduce nutrient load have had some success for P, but N loads have only substantially decreased locally. Where large N load reductions have been achieved, water column N concentrations and algal biomass have fallen. Baltic ecosystem restoration is still hampered by insufficient understanding of basic factors regulating the biogeochemical cycles of N (particularly denitrification and $N_2$-fixation) and P. But even with this information available, it would be a major challenge to model the ecosystem effects of proposed nutrient management options and to devise cost-effective management procedures with good stakeholder involvement and acceptance.

## ACKNOWLEDGEMENTS

This is a contribution from the research programs Sustainable Coastal Zone Management and Marine Research on Eutrophication of the Swedish Foundation for Strategic Environmental Research (MISTRA). The Stockholm Marine Research Centre provided Fig. 1.

| | | |
|---|---|---|
| Received: | July | 20, 2001 |
| Revised: | October | 8, 2001 |
| Accepted: | October | 10, 2001 |
| Published: | October | 26, 2001 |
